# CD200 Receptor Restriction of Myeloid Cell Responses Antagonizes Antiviral Immunity and Facilitates Cytomegalovirus Persistence within Mucosal Tissue

**DOI:** 10.1371/journal.ppat.1004641

**Published:** 2015-02-05

**Authors:** Gabrielle Stack, Emma Jones, Morgan Marsden, Maria A. Stacey, Robert J. Snelgrove, Paul Lacaze, Laura C. Jacques, Simone M. Cuff, Richard J. Stanton, Awen M. Gallimore, Tracy Hussell, Gavin W. G. Wilkinson, Peter Ghazal, Philip R. Taylor, Ian R. Humphreys

**Affiliations:** 1 Institute of Infection and Immunity, School of Medicine, Cardiff University, Heath Park, Cardiff, United Kingdom; 2 Leukocyte Biology Section, National Heart and Lung Institute, Imperial College London, London, United Kingdom; 3 Division of Pathway Medicine and Edinburgh Infectious Diseases, University of Edinburgh, Edinburgh, United Kingdom; 4 Manchester Collaborative Centre for Inflammation Research (MCCIR), University of Manchester, Manchester, United Kingdom; 5 Wellcome Trust Sanger Institute, Hinxton, Cambridgeshire, United Kingdom; Oregon Health Sciences University, UNITED STATES

## Abstract

CD200 receptor (CD200R) negatively regulates peripheral and mucosal innate immune responses. Viruses, including herpesviruses, have acquired functional CD200 orthologs, implying that viral exploitation of this pathway is evolutionary advantageous. However, the role that CD200R signaling plays during herpesvirus infection *in vivo* requires clarification. Utilizing the murine cytomegalovirus (MCMV) model, we demonstrate that CD200R facilitates virus persistence within mucosal tissue. Specifically, MCMV infection of CD200R-deficient mice (CD200R^-/-^) elicited heightened mucosal virus-specific CD4 T cell responses that restricted virus persistence in the salivary glands. CD200R did not directly inhibit lymphocyte effector function. Instead, CD200R^-/-^ mice exhibited enhanced APC accumulation that in the mucosa was a consequence of elevated cellular proliferation. Although MCMV does not encode an obvious CD200 homolog, productive replication in macrophages induced expression of cellular CD200. CD200 from hematopoietic and non-hematopoietic cells contributed independently to suppression of antiviral control *in vivo*. These results highlight the CD200-CD200R pathway as an important regulator of antiviral immunity during cytomegalovirus infection that is exploited by MCMV to establish chronicity within mucosal tissue.

## Author Summary

Immune inhibitory receptors, including CD200 receptor (CD200R), can limit immune responses in the mucosa to restrict reactivity to the plethora of harmless antigens that mucosal surfaces are continually exposed to. However, viruses may exploit these suppressive mechanisms to enable their persistence and spread. Many viruses, including herpesviruses, have acquired functional homologs of CD200, the ligand of CD200R, implying that viral exploitation of this pathway is evolutionary advantageous. We now show that the β-herpesvirus murine cytomegalovirus (MCMV) takes advantage of the CD200R inhibitory pathway to persist within a mucosal site of MCMV persistence, the salivary glands. Mice deficient in CD200R mounted elevated antiviral immune responses that were driven by the increased division and accumulation of myeloid cells that function to orchestrate the generation of antiviral effector immune responses. Interestingly, MCMV infection of myeloid cells up-regulated CD200 expression. Thus, MCMV exploits the CD200 pathway to persist within mucosal tissue.

## Introduction

CD200R is an Immunoglobulin superfamily family member that is expressed by hematopoietic cells, with notably high expression on myeloid cells [1]. The ligand of CD200R, CD200 (OX2), is broadly expressed by cells of hematopoietic and non-hematopoietic origins [2]. The primary function of the CD200R pathway is to limit immune reactivity. CD200-CD200R interactions induce a unidirectional inhibitory signal within CD200R-bearing cells that is mediated by tyrosine motifs in the cytoplasmic domain of CD200R that recruit DOK2 and RasGAP, resulting in inhibition of the ERK pathway [3–6].

The CD200R pathway negatively regulates myeloid cell homeostasis in the periphery [7], and in the pulmonary [8] and, to a lesser extent, the intestinal [9] mucosa. CD200R signaling limits the rapid onset of experimental autoimmune encephalomyelitis [3, 7] and restrains bacterial-induced inflammation [10]. Importantly, CD200R also restricts viral-induced inflammation during respiratory influenza infection [9, 11] and herpes simplex virus (HSV) infection of the cornea [11]. However, CD200R also restricts IFN-dependent control of corona virus infection via regulation of TLR7 [12] and control of intracranial HSV infection [13], demonstrating that this inhibitory receptor can impinge on protective antiviral immunity.

During evolution, numerous herpesviruses have acquired proteins with the potential to induce immune inhibitory receptor signaling [14]. For example, human cytomegalovirus (HCMV) encodes a functional homolog of the inhibitory cytokine interleukin-10 (IL-10) [15]. Rhesus CMV lacking its IL-10 homolog induces increased virus-specific immune responses [16], and IL-10R signaling during murine cytomegalovirus (MCMV) infection antagonizes antiviral immunity and facilitates virus persistence [17–19]. Thus, these studies provide *in vivo* experimental evidence supporting a rationale for CMV exploitation of host immune regulatory pathways.

Intriguingly HCMV UL119–121 proteins display homology to human CD200 [20], although it is currently unknown whether they induce inhibitory signaling through CD200R. However, numerous herpesviruses are known to encode functional CD200 orthologs (vCD200s) implying that exploitation of this inhibitory pathway is potentially advantageous for herpesviruses. The most well-characterized vCD200 is the Kaposi’s sarcoma-associated herpesvirus (KSHV) protein K14, which suppresses the activation of neutrophils [21], basophils and NK cells [22], T cells [23] and macrophages [24] *in vitro*. Furthermore, the English isolate of rat cytomegalovirus (RCMV-E) encodes a CD200 homolog (e127) capable of binding CD200R [25, 26].

Despite the possible importance of the CD200-CD200R pathway in modulating anti-CMV immunity, how it influences antiviral immune responses and virus replication during infection *in vivo* requires clarification. To investigate this, we studied MCMV infection in wild type mice and mice lacking CD200R. Experiments revealed a pivotal role for CD200R regulation of myeloid cell responses in limiting antiviral CD4 T cell responses. We provide evidence that MCMV exploits the CD200-CD200R pathway to facilitate persistent infection within mucosal tissue.

## Results

### CD200R promotes MCMV persistence in the salivary glands

**Figure 1:**
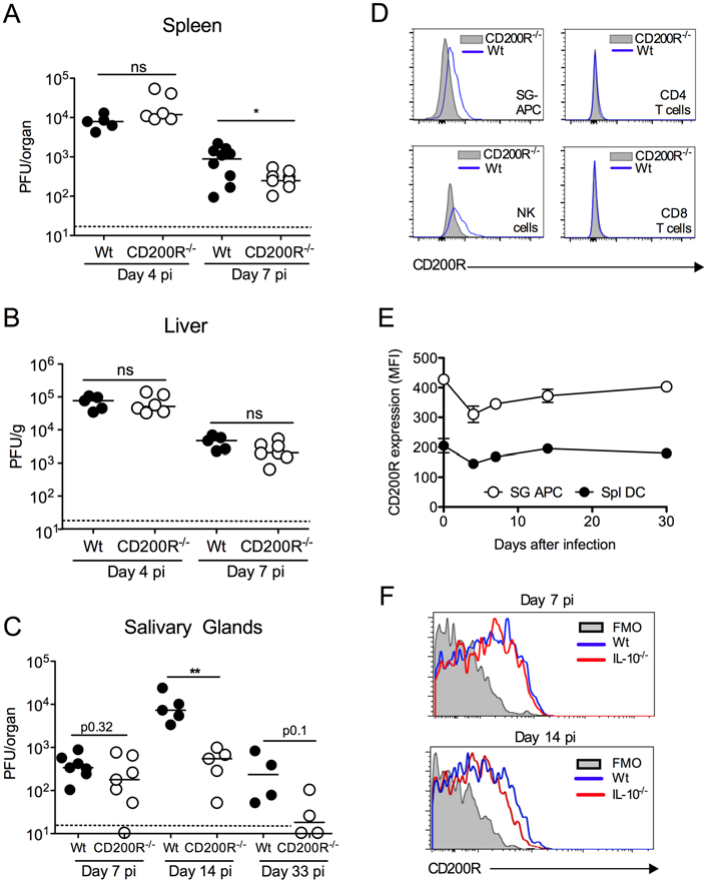
CD200R promotes MCMV persistence in the mucosa. (A-C) MCMV titres in the spleen (A), liver (B) and SGs (C) were measured 4 and 7 (A&B) or 7, 14 and 33 (C) days pi. Individual mice and median are shown. Horizontal dashed lines depict the lower limit of detection. (D) Histogram overlays of CD200R expression by SG leukocytes at day 7 pi. Blue line = wt, shaded = CD200R^-/-^. (E&F) Intensity of surface CD200R expression by APCs (E) isolated from the SGs and spleens of wt mice is represented as median fluorescent intensity (MFI). Results represent the mean +/- SEM of 3 mice. (F) Wt and IL-10^-/-^ mice were infected with MCMV and on 7 or 14 days pi CD200R expression by SG-APCs was analyzed by flow cytometry. Fluorescence Minus One (FMO) control = SG-APCs from infected wt mice 7 (left) or 14 (right) days pi. Plots are representative of 3–4 mice. All data is representative of 2–3 separate experiments.

MCMV replicates in numerous organs, including the spleen, liver and lungs, during acute infection, prior to dissemination to the salivary glands (SGs), in which MCMV replicates for 1–2 months [27, 28]. We hypothesized that CD200R signaling may facilitate MCMV replication *in vivo*. To test this, wild type C57BL/6 (wt) and CD200R^-/-^ mice were infected with MCMV and virus load measured. Peak acute MCMV replication at day 4 post-infection (pi) in the spleen (Fig. 1A) and liver (Fig. 1B) was unaltered by CD200R deficiency. However, CD200R^-/-^ mice exhibited a reduced burden of replicating virus (Fig. 1A) in the spleen 7 days pi.

We next investigated whether CD200R promoted MCMV persistence. In our model, replicating virus is first detectable in the SGs at day 7 pi. Virus load in wt and CD200R^-/-^ mice day 7 pi was comparable (Fig. 1C), suggesting that improved antiviral control in spleens of CD200R^-/-^ mice (Fig. 1A) did not influence dissemination to the SGs and associated brown fat in which MCMV replicates at this time-point [29]. Crucially, however, CD200R^-/-^ mice restricted persistent MCMV replication in the SGs 14 days pi, and more CD200R^-/-^ mice cleared MCMV by day 33 pi as compared to wt controls (Fig. 1C). Thus, intact CD200R during chronic infection promoted virus persistence within this mucosal organ.

### CD200 and CD200R are expressed during MCMV infection

Consistent with biological impact of CD200R within the SGs, we observed significant CD200R expression by CD11c^+^MHC II^+^ salivary gland (SG) APCs (referred to hereafter as SG-APCs, Fig. 1D&E), which are phenotypically indicative of tissue-resident macrophages [30], and NK cells (Fig. 1D) but not CD4 and CD8 T cells (Fig. 1D). CD200R expression by SG myeloid cells was notably higher than splenic counterparts (Fig. 1E), demonstrating enhanced expression of CD200R in mucosal versus non-mucosal sites of MCMV infection. CD200R expression by myeloid cells in both compartments was relatively stable during infection, with a slight reduction in the intensity of CD200R expression 4 days pi prior to recovery to steady-state levels by 14 days (Fig. 1E). Interleukin-10 (IL-10) is expressed in the SGs in response to MCMV infection and promotes virus persistence [18, 31]. Although IL-10 induces CD200R expression by macrophages *in vitro* [8], MCMV-infected IL-10^-/-^ mice exhibited no alterations in CD200R expression by myeloid cells during infection (Fig. 1F). Thus, CD200R was expressed during infection but was not significantly upregulated in response to MCMV, by either an IL-10-dependent or independent mechanism.

**Figure 2:**
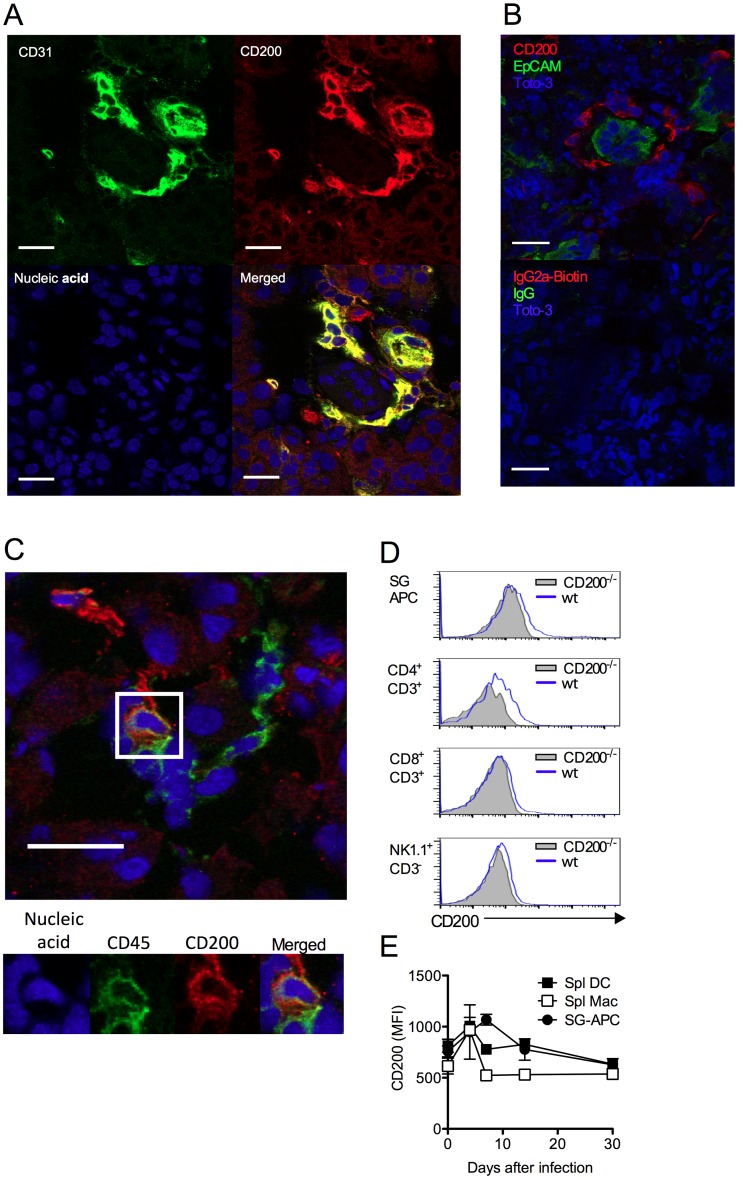
CD200 is expressed during *in vivo* MCMV infection. (A-B) Wt mice were infected with MCMV and SGs harvested 7 days pi. (A) CD31 (green) and CD200 (red) co-localization in SGs. (B) Distinct CD200 (red) expression from EpCAM^+^ (green) acinar epithelial cells (top). Isotype controls for CD200 (Rat IgG2a-Biotin) and EpCAM (Rabbit IgG), and the secondary antibodies Streptavidin 555 and Alexa Fluor 488 anti-rabbit IgG (Bottom). (C) Hematopoietic cell (green [CD45]) expression of CD200 (red). (A-C) Magnification = 63x, white scale bars = 20μm. (D) Representative histogram overlays of CD200 expression by SG-APCs, CD4 and CD8 T cells and NK cells in the SGs at day 7 pi. FMO controls are shown from cells isolated at day 7 pi. (E) CD200 expression by SG and splenic myeloid cell populations was assessed over time. Splenic DCs: CD11c^+^MHC II^+^; splenic macs: F4/80^+^CD11b^+^. Data is represented as mean ± SEM of 3 mice/group representing 4 experiments.

Unlike certain herpesviruses [14, 24], MCMV does not encode an obvious vCD200 [32]. Within infected SGs, CD200^+^ cells were predominantly large CD31^+^ cells (Fig. 2A, isotype controls:S1A Fig.) that were EpCAM^-^ (Fig. 2B), suggestive of endothelial cell origin, and not EpCAM^+^ acinar epithelial cells in which MCMV replicates during the persistent phase of infection [33]. CD200^+^ cells did not express alpha smooth muscle actin (S1B Fig.), also demonstrating these cells were not myoepithelial cells. Interestingly, CD200^+^ cells were often observed in ring-like structures around acinar epithelial cells (Fig. 2B), indicative of capillary networks that surround acini [34]. CD200^+^CD31^+^ cells were detectable in naïve SGs (S1C Fig.), and we observed no notable increase in the intensity of CD200 expression by CD31^+^ cells within infected tissue. In addition to abundant CD200^+^CD31^+^ cells, a more scarce population of CD200^+^CD45^+^ cells was also detectable within the SGs indicating the presence of CD200 on a hematopoietic cell type(s) (Fig. 2C). Analysis by flow cytometry revealed significant CD200 expression by APCs and T cells within the SGs and spleen (Fig. 2D&E) during MCMV infection.

### MCMV infection of macrophages up-regulates CD200

**Figure 3:**
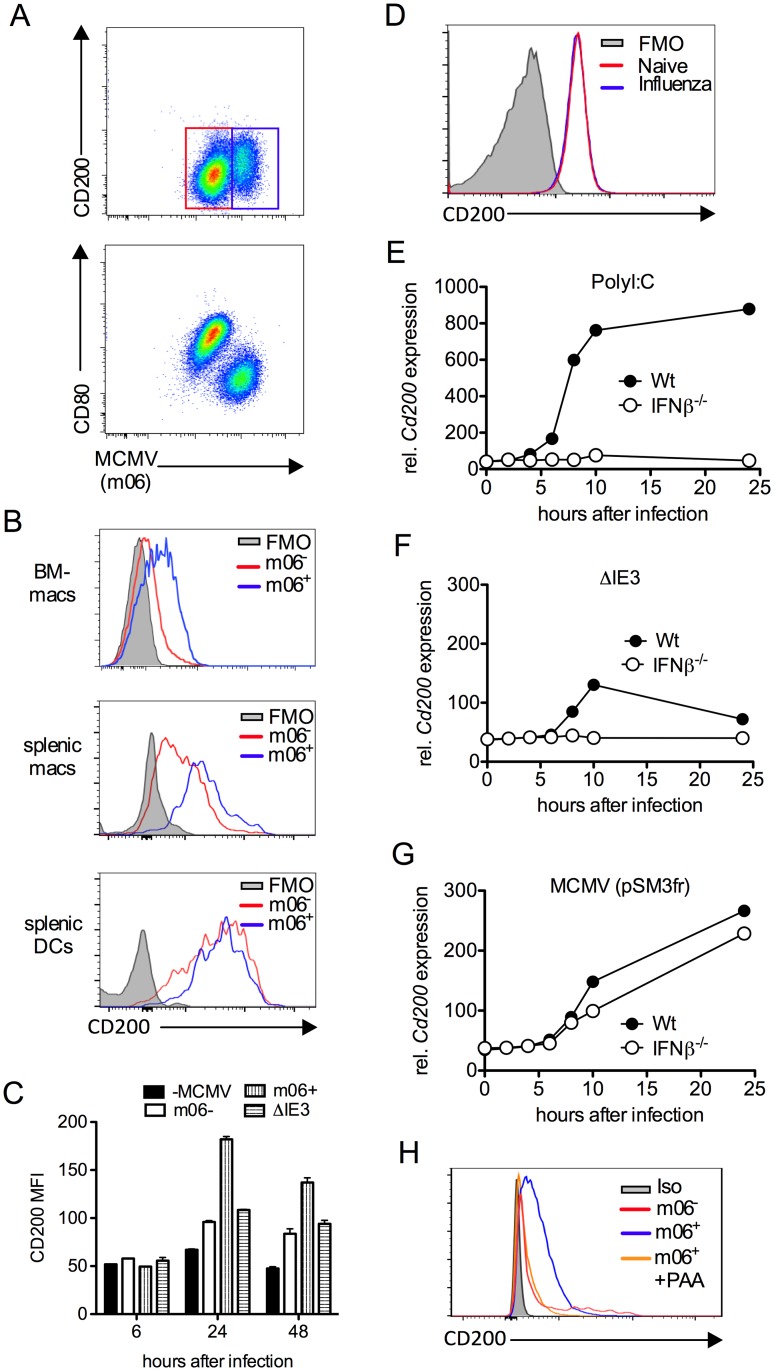
MCMV induces CD200 expression by macrophages. (A-C) Bone marrow-derived macrophages (BM-DMs) were infected with pSM3fr MCMV and CD200 expression was assessed. (A) Representative bivariant flow cytometry plots of MCMV infection (m06^+^) versus CD200 (top) and CD80 (bottom) 24 hrs pi. (B) Representative histograms of CD200 expression by infected (m06^+^, blue) and uninfected/virus exposed cells from the same well (m06^-^, red). BM-DMs (top), splenic F4/80^+^ macrophages (middle) and splenic CD11c^hi^ MHC II^hi^ DCs (bottom). FMOs are cells from infected cell cultures (containing infected and uninfected cells). (C) CD200 expression by MCMV-infected (m06^+^), MCMV-exposed (m06^-^) and _Δ_IE3 MCMV-infected BM-DMs was compared to expression by mock-infected cells. Data are expressed as median fluorescence intensity (MFI) and are expressed as mean + SEM of 2 replicates. Results represent 2–5 experiments. (D) BM-DMs were infected or not with influenza strain PR8 (MOI:1), and CD200 expression was assessed 24hrs later by flow cytometry. FMOs are from uninfected macrophages. Data from 1 of 2 experiments is shown. (E-G) BM-DMs were treated with (E) PolyI:C or infected with (F) replication deficient _Δ_IE3 MCMV or (G) wt MCMV (pSM3fr) and *cd200* expression assessed by microarray. (H) BM-DMs were infected with wt MCMV +/- PAA, and CD200 expression was assessed 24hrs later. Data is shown as a representative histogram (of 2 separate experiments) depicting CD200 expression by uninfected/virus exposed cells (m06^-^, red), infected cells (m06^+^, blue) and PAA-treated m06^+^ cells (orange). Grey = m06^+^ untreated cells stained with isotype control (rat IgG2a-PE).

Interestingly, we noted that CD200 expression by APC populations in the SGs and spleen was induced above baseline upon infection (Fig. 2D&E). We hypothesized that MCMV infection of myeloid cells may directly influence CD200 expression. We infected myeloid cell populations *in vitro* using a multiplicity of infection of 1 that leads to an infection efficiency of less than 60% (see Fig. 3A for example), enabling us to compare surface CD200 protein levels on uninfected and infected cells from the same well of a tissue culture plate, as identified by flow cytometric detection of the intracellular MCMV m06 protein. Infection of bone marrow-derived macrophages (BM-DM, Fig.3A–C) and splenic macrophages (Fig.3B) up-regulated CD200 (Fig.3A–C). Importantly, we observed a marked increase in CD200 expression by infected (m06^+^) as compared with uninfected (m06^-^) macrophages derived from the same wells (Fig. 3A–C), suggesting that CD200 is preferentially up-regulated by macrophages in which MCMV is actively replicating.

Infection of BM-DM with influenza did not trigger CD200 expression (Fig. 3D), demonstrating that CD200 up-regulation is not a generic macrophage response to viruses. However, CD200 expression is induced by ligation of TLRs, including TLR3 and TLR9 [10]; both of which are triggered by MCMV [35, 36]. In accordance, TLR3 ligation by PolyI:C induced substantial CD200 mRNA expression by macrophages in an IFNβ-dependent manner (Fig. 3E). To investigate whether MCMV induction of *Cd200* transcription required productive replication, we compared expression following macrophage infection with IE3 knockout replication-deficient MCMV (_Δ_IE3)[37] and replication-sufficient wt MCMV (pSM3fr). Macrophage exposure to _Δ_IE3 MCMV induced a small, transient induction of CD200 mRNA in an IFNβ-dependent manner (Fig. 3F), consistent with TLR-mediated induction of CD200 triggered during incomplete MCMV replication, and the moderate CD200 protein expression by uninfected (m06^-^) macrophages derived from infected cell cultures (Fig. 3C). In contrast, replicating MCMV induced substantial and prolonged CD200 mRNA expression independently of IFNβ (Fig. 3G). Furthermore, inhibition of viral DNA polymerase with phosphonoacetic acid (PAA) antagonized MCMV-induced CD200 expression in BM-DMs (Fig. 3H), again demonstrating the requirement for productive virus replication in this process. Importantly, we observed that SG-APCs did not support MCMV replication *in vitro* in accordance with the absence of detectable infection *in vivo* [38], and MCMV infection of splenic DCs did not further induce CD200 expression (Fig. 3B). Thus, these data suggested that myeloid cells up-regulated CD200 during MCMV infection and, in the case of macrophages in secondary lymphoid tissues, MCMV induces CD200 expression independently of TLR stimulation during productive replication.

### CD200 derived from hematopoietic and non-hematopoietic cells promotes MCMV persistence

**Figure 4:**
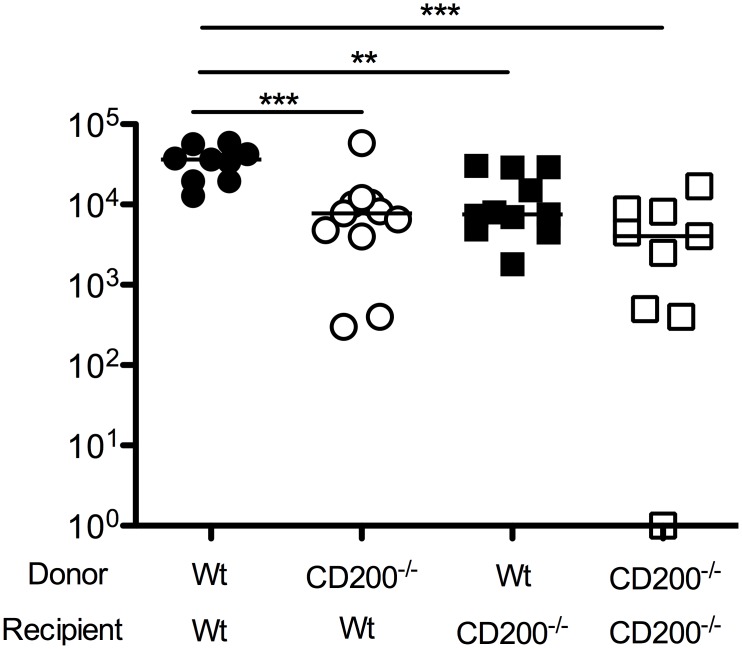
CD200 expressed by hematopoietic and non-hematopoietic cells facilitate MCMV persistence. Mixed wt/CD200^-/-^ bone marrow chimeras were generated and infected with MCMV. 14 days later, replicating virus load in the SGs was assessed by plaque assay. Individual mice and median are shown. Horizontal dashed line depicts the lower limit of detection. Data from 3 merged experiments is shown. Similar results were observed in a fourth experiments but data was omitted due to inter-experimental variation—median values for all groups were ~1 log lower than medians of data depicted in the Figure.

Given that CD200 expression by both hematopoietic and non-hematopoietic cells was observed in MCMV-infected mice, we sought to understand which cellular compartment was responsible for inhibiting antiviral immunity. We made bone marrow chimeras derived from wt mice or mice deficient of CD200, generating mice lacking CD200 within the hematopoietic and/or radiation-resistant (non-hematopoietic) compartment. We then studied virus load in SGs 14 days post-MCMV infection. Interestingly, deleting CD200 from either compartment reduced virus load as compared to wt>wt mice (Fig. 4), demonstrating that CD200 expressed by hematopoietic and non-hematopoietic cells both delivered immune suppressive signals that promoted MCMV persistence.

### CD200R restricts myeloid cell accumulation and proliferation

**Figure 5:**
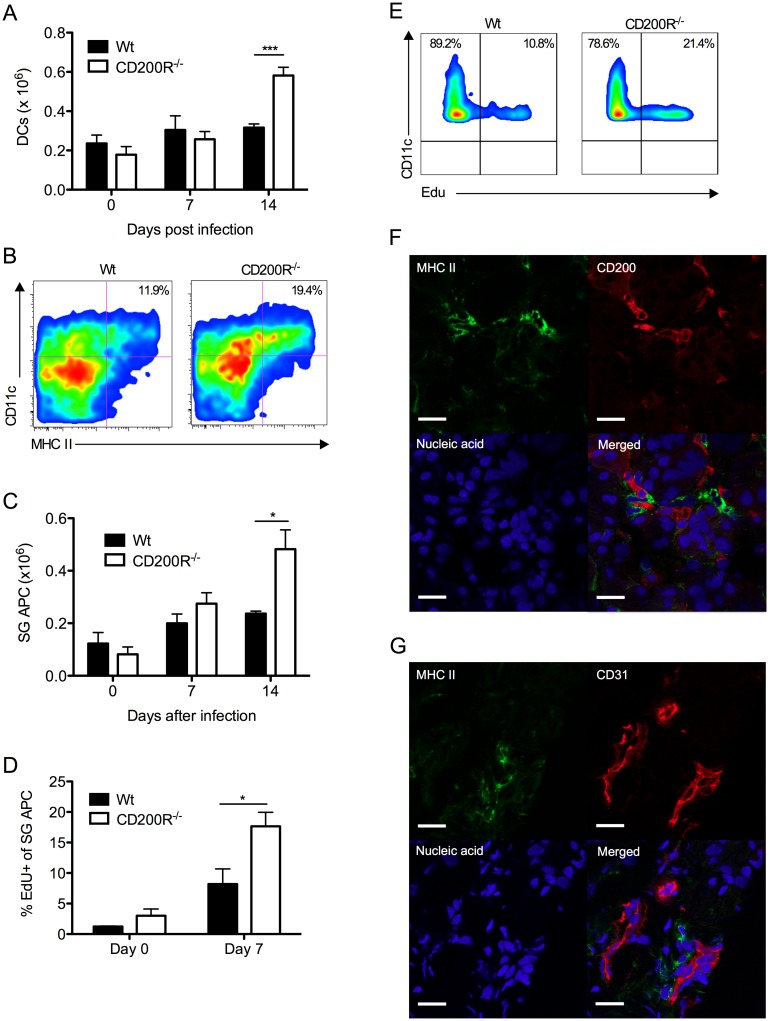
CD200R restricts myeloid cell accumulation and SG-APC proliferation. (A) Time-course of accumulation of splenic CD11c^+^MHC II^+^ cells in wt and CD200R^-/-^ mice. Mean + SEM of 4–11 mice/group is shown. (B) Representative flow cytometry plots of CD11c^+^MHC II^+^ SG-APCs in wt (left) and CD200R^-/-^ (right) at day 7 pi. (C) Numbers of SG-APCs over time. Mean + SEM of 6 mice/group is shown. (D) Proliferating (EDU^+^) SG-APCs were assessed by flow cytometry. Mean + SEM of 3–6 mice/group is shown. (E) Representative bivariant flow cytometry plots of EDU incorporation by SG-APCs in wt (left) or CD200R^-/-^ (right) mice at day 7 pi. Data is gated on live CD11c^+^MHC II^+^ cells. All results represent at least 2 independent experiments. (F&G) Wt mice were infected with MCMV and SGs harvested 7 days pi. (F) MHC II (green) expressing cells adjacent to large CD200^+^ (red) endothelial cells. (G) MHC II (green) expressing cells adjacent to CD31^+^ (red) endothelial cells. Sections were counterstained with TOTO-3 (blue) to detect DNA. Magnification = 63x, white scale bars = 20μm.

We assessed the impact of CD200R deficiency on virus-induced myeloid cell responses. Reduced MCMV persistence in CD200R^-/-^ mice was accompanied by accumulation of splenic DCs 14 days pi (Fig. 5A). The inability of SG-APCs to cross-present antigen to CD8 T cells (in combination with MCMV down-regulation of MHC class I) has been shown to be responsible for the lack of CD8 T cell-mediated control of MCMV replication in the SGs, demonstrating that local antigen-presenting function of SG-APCs is a critical determinant of protective T cell immunity during MCMV persistence [38]. Interestingly, SG-APC accumulation in infected CD200R^-/-^ mice was substantially increased 14 days pi (Fig. 5B&C). However, SG-APC numbers were comparable in wt and CD200R^-/-^ mice following resolution of MCMV infection in our model (48 days pi, S2A Fig.). Thus, CD200R restricted mucosal myeloid cell accumulation during early time-points of SG infection rather than influencing myeloid cell turnover during the resolution phase of infection.

Tissue resident macrophages proliferate in response to inflammatory stimuli [39, 40]. Thus, we measured SG-APC proliferation before and after MCMV infection 7 days pi. Low levels of SG-APC homeostatic proliferation were measured in naïve wt and CD200R^-/-^ mice (Fig. 5D). However, infection-induced SG-APC proliferation was further elevated in CD200R^-/-^ mice as compared to wt mice day 7 pi (Fig. 5D&E), a time at which CD200R was expressed by these cells in wt mice (Fig. 1D&E). This suggested that increased SG-APC accumulation in CD200R^-/-^ mice was a consequence of heightened proliferation. Importantly, visualization of MHC II^+^ cells within the SGs revealed that MHC II^+^ cells were consistently located adjacent to large CD200^+^ cells 7 days (Fig. 5F) and 14 days (S2B Fig.) pi. In accordance with CD200 expression by CD31^+^ cells (Fig. 2A), MHC II^+^ cells were also observed surrounding CD31^+^ vessels (Fig. 5G), suggesting that tissue-resident MHC II^+^ SG-APC interactions with CD200-bearing endothelial cells restricts infection-induced cellular proliferation. In support of this conclusion, chimeric mice lacking CD200 only in non-hematopoietic cells exhibited increased SG-APC accumulation (S2C Fig.). Furthermore, improved control of MCMV in these mice (Fig. 4) in addition to the absence of an impact of non-hematopoietic cell-derived CD200 on splenic DC responses (S2D Fig.) points towards a role for local SG-APC expansion in determining control of MCMV replication in the mucosa.

### CD4 T cells limit virus persistence in CD200R^-/-^ mice

**Figure 6:**
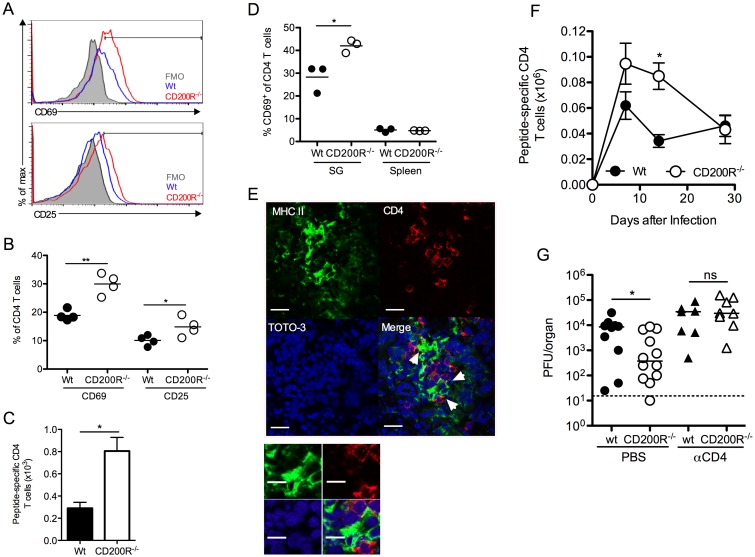
CD4 T cells limit virus persistence in CD200R^-/-^ mice. (A-B) CD69 and CD25 expression by SG CD4 T cells was assessed 10 days pi by flow cytometry and data depicted as histogram overlays (A) and percent expression by individual mice + mean (B). FMO in (A) was derived from d10 infected CD200R^-/-^ mice. (C) MCMV-specific SG CD4 T cells were isolated 14 days pi, stimulated with pooled MCMV-derived peptides (m09, M25, M139 and m142) and peptide-reactive IFNγ^+^ cells were enumerated by flow cytometry. Results represent the mean and SEM of 4–6 mice/group, representing 2 experiments. (D) CD69 expression by CD4 T cells from the SGs and spleen at 14 days pi was determined by flow cytometry. Percent CD69 expression of individual mice + mean is shown. (E) Co-localization of MHC class-II expressing cells (green) with CD4 T cells (red) in the SGs 7 days pi. White arrows indicate co-localization of CD4 T cells with MHC II^+^ cells. Magnification = 63x, scale bars = 20μm. (F) Virus-specific CD4 T cells were enumerated in the spleen over a time-course as described in (C). Results represent the mean and SEM of 4–6 mice/group, representing 2 experiments. (G) Wt and CD200R^-/-^ mice were infected with MCMV and treated with αCD4 depleting antibodies or PBS control at days 4 and 6 pi. SGs were harvested 14 days pi and virus load assessed by plaque assay. Horizontal dashed line depicts the lower limit of detection. Individual mice and median are shown and data is representative of 2 experiments.

CD4 T cells are critical effector cells in the control of MCMV persistence that afford protection via expression of IFNγ [38, 41]. Despite the absence of measurable T cell expression of CD200R (Fig. 1D), SG-infiltrating CD4 T cells in CD200R^-/-^ mice exhibited increased activation, indicated by CD69 and CD25 up-regulation 10 days pi (Fig. 6A&B). Enrichment of CD25^hi^ CD4 T cells were not observed in either wt or CD200R^-/-^ mice (Fig. 6A), consistent with the absence of regulatory T cell infiltration into the SGs in response to MCMV [31]. Importantly, IFNγ^+^ virus-specific CD4 T cell numbers were elevated in the SGs of CD200R^-/-^ mice by 14 days pi (Fig. 6C). In addition to activated T cells, CD4^+^ tissue-resident memory T cells also express CD69 [42]. Interestingly, whereas CD69 expression by SG CD4 T cells was elevated in CD200R^-/-^ mice 14 (Fig. 6D) and 30 (S3A Fig.) days pi, elevated prolonged expression of CD25 by CD200R^-/-^ SG CD4 T cells was not observed (S3B Fig.), implying that CD200R may also restrict the accumulation and/or retention of CD4 T cells with a tissue-resident memory-like phenotype. Furthermore, MHC II^+^ SG-APCs that proliferate and accumulate to higher numbers in CD200R^-/-^ mice (Fig. 5B–E) co-localized with CD4 T cells (Fig. 6E), suggesting that elevated myeloid cell responses within the SGs of CD200R^-/-^ mice enhanced mucosal CD4 T cell responses. In addition, elevated splenic DC numbers 14 days pi in CD200R^-/-^ mice (Fig. 5A) were accompanied by an increase in virus-specific CD4 T cells in this organ at this time (Fig. 6F). These data therefore suggested that CD200R restricted peripheral and mucosal CD4 T cell responsiveness during virus persistence through localized regulation of tissue APC accumulation.

We next investigated whether elevated CD4 T cell responses restricted MCMV persistence in CD200R^-/-^ mice. Depletion of CD4 T cells abrogated the improved control of MCMV in CD200R^-/-^ mice (Fig. 6G), which is consistent both with the established role for CD4 T cells in limiting MCMV persistence in the SGs [38, 41], and the conclusion that MCMV exploits CD200R to facilitate persistence predominantly by antagonizing proliferation and accumulation of MHC class II-bearing myeloid cells.

## Discussion

We demonstrate that MCMV exploits the CD200-CD200R pathway to restrict mucosal antiviral immunity *in vivo* to facilitate MCMV persistence in a secretory mucosal organ. Restriction of myeloid cell responses was central to the inhibitory action of CD200R. CD200R signaling limited accumulation of MHC class II-bearing APCs in both the periphery and mucosa thus restricting the ensuing virus-specific CD4 T cell response. CD200R restricted SG-APC responses by limiting virus-induced cellular proliferation. CD200R inhibition of this process has likely evolved to limit responses to harmless antigens that mucosal surfaces are continually exposed to. However our data demonstrate that MCMV benefits from this immune-regulatory pathway to persist within its mammalian host, and MCMV can actively induce CD200 expression during infection.

SG-APCs proliferated in response to MCMV infection, consistent with the ability of tissue-resident macrophages to undergo a proliferative burst following inflammation [39, 40, 43]. Our data suggest that the interaction of SG-APCs with the basal surface of CD200-bearing endothelial cells limits this process, and implies that the large vascular network within the SGs may function not only as a blood supply but also to deliver inhibitory signals that, in the context of homeostatic conditions, functions to limit immune responsiveness. The identification of ring-like structures surrounding acini implies a scenario during MCMV infection in which CD200R-expressing myeloid cells situated close to or migrating towards infected cells may receive inhibitory signals from CD200-expressing vascular structures.

Currently, there are no methodologies available to exclusively delete SG-APCs *in vivo*. Therefore we are unable to make definitive conclusions regarding the function of these cells in our experiments. However, our data supports a model in which CD200R-mediated restriction of SG-APC proliferation reduces CD4 T cell activation within the SGs, subsequently impairing CD4 T cell responsiveness and control of MCMV persistence. Our data also demonstrated that CD200R impaired the accumulation of virus-specific CD4 T cells in the periphery that was accompanied by reduced splenic DC accumulation. Thus, CD200R signaling impinges on antiviral protection from mucosal MCMV replication by restricting CD4 T cell activation and expansion both within the mucosa itself, but also in secondary lymphoid tissue.

Intriguingly, persistent MCMV infection of CD200R^-/-^ mice led to the enrichment of CD69^+^ CD4 T cells not expressing the activation marker CD25. CD69 is expressed by tissue-resident memory CD4 T cells [42]. MCMV replication continued at the time-points at which CD69^+^ CD4 T cells were detected in our study, thus precluding definitive conclusions regarding bone fide tissue-resident memory cells. However, our data implies that CD200R may indirectly restrict the accumulation of CD4 T cells in the SGs that exhibit a phenotype indicative of tissue-resident memory CD4 T cells.

CD200R facilitates early viral replication in acute MHV [12] and HSV [13] infections *in vivo*. In contrast, we observed that early control of MCMV was unaffected by CD200R. This may reflect in part that CD200R deficiency did not influence MCMV replication in macrophages (S4A Fig.), unlike data reported in HSV infection [13]. Improved control of MHV infection in CD200^-/-^ mice was associated with elevated type I IFN [12]. Type I IFN was not measured in our study and may not be altered in MCMV-infected CD200R^-/-^ mice. Also, type I IFN exerts potent antiviral activity against MCMV *in vivo* in wt mice [44] and may therefore be produced at levels that exert maximal antiviral activity in our model irrespective of any impact of CD200R on cytokine expression.

Instead, we show for the first time that CD200R signaling influences persistent virus replication *in vivo*. Improved control of MCMV replication in the SGs in CD200R^-/-^mice was intriguing given that MCMV does not encode an obvious CD200 homolog. This may be explained in part by the existence of a structural CD200 ortholog encoded by MCMV that lacks sufficient sequence similarity to be detected, or by the existence of other viral ligands for CD200R. Importantly however, experiments utilizing CD200^-/-^ mice highlighted a role for cellular CD200 in dampening antiviral immunity. Cellular CD200 restricts virus-induced immune responses in acute virus infections [8, 12, 45], and our data supports the conclusion that some viruses may exploit host CD200-CD200R interactions to establish persistence. Intriguingly, *in vivo* experiments investigating a functional role for CD200 orthologs expressed by RCMV [26] and Rhesus macaque rhadinovirus [46] failed to detect significant benefit of these vCD200s in promoting herpesvirus persistence in these experimental models. Our data suggest the benefit of herpesvirus exploitation of host CD200 expression, irrespective of whether the virus also encodes its own vCD200 protein.

Results obtained from bone marrow chimeras demonstrate the importance of non-hematopoietic cell-derived CD200 in facilitating MCMV persistence, thus supporting an important role for endothelial cells in indirectly restricting antiviral CD4 T cell responses via regulation of myeloid cells. However, a significant role for hematopoietic cells in promoting virus persistence was also revealed in these experiments. Peripheral and mucosal myeloid cells expressed CD200 during MCMV infection. Although SG-APCs did not support MCMV replication, splenic macrophages up-regulated CD200 following direct MCMV infection *in vitro*. MCMV infection of wt and CD200R^-/-^ bone marrow-derived macrophages resulted in comparable expression of MHC II (S4B Fig.), suggesting that MCMV does not exploit macrophage expression of CD200 to deliver an autocrine inhibitory signal; a conclusion further supported by comparable MCMV replication in wt and CD200R^-/-^ macrophages and consistent with the inability of CD200 to interact with CD200R in a *cis*-cellular fashion [47, 48].

Instead our data suggest that a CD200-bearing myeloid cell may restrict antiviral immunity and that, in the case of peripheral infection, MCMV influences this process.

CD200 may suppress myeloid cell activity and/or accumulation indirectly via an unknown CD200R-expressing cell subset, or by directly triggering CD200R signaling within a myeloid cell. T cells expressed CD200 during MCMV infection, implying that MCMV may also passively exploit a negative feedback loop by which CD200-bearing T cells deliver an inhibitory signal to CD200R-bearing myeloid cell with which they interact. Notably however, non-hematopoietic cell-derived CD200 restricted myeloid cell accumulation within the SGs, suggesting that T cells do not exert CD200-mediated inhibition of myeloid cell proliferation within this particular site of MCMV infection. Irrespective of the exact mechanism(s), our data suggest that CD200 expressed by hematopoietic cells impacts on the development of antiviral immunity that subsequently allows virus persistence within the SGs, and that MCMV actively exploits this process.

MCMV induced myeloid cell CD200 expression via two distinct mechanisms. Firstly, incomplete virus replication triggered TLR-induced IFNβ-dependent *Cd200* gene expression. Importantly, replication-competent virus induced *Cd200* expression in macrophages independently of this pathway, and CD200 induction was dependent upon viral DNA polymerase activity. The mechanism through which MCMV actively regulates CD200 is not clear. CMV infection induces profound alterations in host cell protein production and gene expression [49–52]. Concurrent analysis of *Cd200* gene and surface protein expression highlighted that viral induction of CD200 occurred at the transcriptional level. The impact of PAA on virus-induced CD200 expression suggests the involvement of a gene product or products expressed during the latter stages of virus replication. However, this conclusion is guarded given that inhibition of viral DNA polymerase during HCMV infection also incompletely inhibits production of certain viral proteins expressed at early times during the virus life-cycle [53].

Whether a viral gene product(s) directly or indirectly induces CD200 expression and which viral protein is responsible remains to be elucidated. Influenza infection of macrophages did not trigger CD200 expression despite the CD200-CD200R pathway restricting influenza-induced T cell responses *in vivo* [8, 12]. Thus, CD200 induction is not a generic response mounted by macrophages in response to viruses. Instead, our experiments demonstrate that MCMV gene expression is essential for this process and implies that CD200 up-regulation represents a previously unappreciated mechanism exploited by CMV, and perhaps other viruses, to antagonize host antiviral immunity.

Collectively, our study highlights a central role for myeloid cells in modulating cytomegalovirus-specific T cell responses in mucosal tissue and the potential importance of regulation of tissue-resident macrophage proliferation in this process. Our study also points towards the manipulation of cellular CD200 expression as a mechanism through which herpesviruses evade host immunity, suggesting that MCMV exploits CD200R signaling to antagonize myeloid cell orchestration of antiviral immunity to promote persistence within and dissemination from the mucosa.

## Materials and Methods

### Mice, viral infections and treatments

C57BL/6 experimental mice were obtained from Harlan UK. CD200R^-/-^ mice were originally generated and provided by Reginald Gorczynski (University Health Network, Toronto), and David Copland (University of Bristol) provided the OX-2^-/-^ mice, with kind permission from Jonathon Sedgwick (Eli Lilly, Indianapolis). IL-10^-/-^ mice were purchased from Jackson Laboratories and maintained in-house.

MCMV Smith strain (ATCC) was prepared in BALB/c salivary glands and purified over a sorbital gradient. Mice were infected by the intra-peritoneal route (i.p) with 3 x 10^4^ PFU MCMV. Some mice were injected i.p with 200µg αCD4 antibody (100µg clone YTS191, 100µg clone YTS3) on days 4 and 6 pi. To measure proliferation *in vivo*, mice were injected i.p with 1mg/mouse EdU (Life Technologies) at day 6 pi. To generate chimeric mice, recipients were irradiated at 2 x 550G, transfused intra-venous (i.v) with 1 x 10^6^ bone marrow cells 24 hours later. Mice were then treated for 3 weeks with baytril-supplemented water. Mice were infected with MCMV 8 weeks after irradiation.

### Ethics statement

All experiments were conducted according to the UK Home Office guidelines at the designated facility at Heath Park, Cardiff University under UK Home Office-approved project licenses PPLs 30/2442 and 30/2969.

### Leukocyte isolation and flow cytometry

SGs and spleens were surgically excised from mice that were euthanized with carbon dioxide. SGs were cut into small pieces and incubated in RPMI 1640 medium (Invitrogen) supplemented with 5 mM CaCl_2_, 5% FCS (Invitrogen), 1 mg/ml collagenase D (Roche Diagnostics), and 10 mg/ml DNAse I (Sigma) at 37°C for 45 minutes, before passing through a cell strainer prior to red blood cell lysis. Leukocytes were then stained with Live/Dead (Invitrogen) prior to incubation with Fc block (eBioscience). Lymphocytes were then stained with a combination of αCD3e-PerCP (Clone 145.2C11, Biolegend), αF4/80-Pacific-Blue (Clone BM8; Biolegend), αIA/IE-PerCP-Cy5.5 (Clone M5/114.15.2, BioLegend), αCD11c-PeCy7 (Clone N418, Biolegend), αNK1.1-allophycocyanin (Clone PK136, BD Biosciences), αCD4-Pacific-blue (Clone RM4.5, BD Biosciences), αCD25-APC-Cy7 (Clone PC61, Biolegend) and αCD69-FITC (Clone H1.2F3, eBioscience).

To detect EdU incorporation, cells were stained as above, fixed with 4% PFA, permeabilized with Saponin buffer, and EdU was labeled with Alexa Fluor 647 using the Click-iT Plus EdU Alexa Fluor 647 Flow Cytometry Assay Kit (Life Technologies) as per manufacturer’s protocol. To detect MCMV-specific CD4 T cells, leukocytes were incubated with 3μg MCMV peptides (Genscript) listed in Figure legends for 6 hours, with BFA (Sigma) for the final 4 hours. CD4 T cells stained as above were permeabilized prior to staining with αIFNγ FITC (clone XMG1.2, eBioscience).

All data were acquired on a BD FACS Canto II. Electronic compensation was performed with antibody-capture beads (BD Biosciences). Data was analyzed using FlowJo software version 10.0.3 (TreeStar Inc, Ashland, OR). Total numbers of different cell populations were calculated by multiplying % positive viable cells detected by flow cytometry x the total number of viable leukocytes (assessed by trypan blue exclusion).

### *In vitro* macrophage infections

Femurs were surgically excised from wt and CD200R^-/-^ mice, sterilized in 70% ethanol and washed in PBS. Bone marrow was isolated, cells centrifuged, washed in RPMI and passed through a 40µM cell strainer. Cells were incubated at 2 x 10^5^ cells/well in D10 media supplemented with 20ng/ml of M-CSF (Peprotech) for 7 days, replenishing M-CSF after 3 days. Spleens and SGs were processed as previously described, with an additional Percoll (GE Healthcare) purification step for SGs after processing. Bone-marrow derived macrophages were infected with MCMV or influenza (PR8) at an MOI of 1. Some cells were also incubated with 300μg/ml phosphonoacetic acid (PAA, Sigma-Aldrich) for 1 hour prior to infection. Splenocytes (2 x 10^5^ cells/well) and SG leukocytes (2 x 10^4^ cells/well) were infected in 48-well plates and infected with MOI 0.5 MCMV. After 24hrs, all macrophages were gently scraped gently off the bottom of the wells, stained with Live/Dead® fixable aqua dead cell stain (Invitrogen) and Fc block (eBioscience), and surface stained with αCD200-PE (Clone OX-90, Biolegend), αCD80 Pacific blue (Clone 16–10A1, Biolegend), αCD86 FITC (Clone GL-1, BD Pharmingen), and αIA/IE PerCP/Cy5.5 (Clone M5/114.15.2, Biolegend) prior to permeabilization and staining with anti-m06 antibody (a kind gift from Stipan Jonjic, Rijeka) conjugated with APC (Innova Biosciences).

### Immunofluorescence

SGs were frozen in OCT and 5μm thick sections fixed in acetone. Sections were blocked with Avidin/Biotin Blocking Kit (Vectorlabs) and then with 2.5% Normal Horse Serum (Vectorlabs). Sections were incubated overnight at 4°C in the dark with CD31-Biotin (Clone MEC 13.3, BD Pharmingen) or CD200-Biotin (Clone OX-90, BioLegend), and MHC II-FITC (Clone M5/114.15.2, BioLegend) or EpCAM (Clone E144, AbCam). Alexa Fluor 488 anti-rabbit IgG (Invitrogen) and Streptavidin Alexa Fluor 555 conjugate (Invitrogen) were used as secondary stains for EpCAM, and CD200-Biotin and CD31-Biotin, respectively. Sections were counterstained with TOTO-3 (Invitrogen), then fixed with 1% PFA and treated with 0.3M glycine. To investigate CD200 colocalization with CD31 or CD45, and MHC II colocalization with CD4, sections were incubated with CD45 (Clone 30-F11, Biolegend), CD31-FITC (Clone 390, eBioscience) or CD4 (Clone RM4–5, Biolegend) overnight at 4°C in the dark. Alexa Fluor 488 anti-rat IgG (Life Technologies), FITC anti-rat IgG2b antibody (Biolegend) and Alexa Fluor 568 goat anti-rat (Life Technologies) were used as secondary stains for CD31-FITC, CD45 and CD4, respectively. Sections were fixed in 1% PFA and treated with 0.3M glycine and then incubated with anti-CD200-Biotin for 2 hours at room temperature, followed by Streptavidin Alexa Fluor 555 conjugate (Invitrogen), or MHC II-FITC (without secondary antibody). Sections were counterstained with TOTO-3 (Invitrogen) and fixed. The following isotype controls were used: Rat IgG2a-Biotin (BD Pharmingen) for CD200-Biotin and CD31-Biotin, Rat IgG2a-FITC (eBioscience) for CD31-FITC, Rat IgG2b-FITC (eBioscience) for MHC II-FITC, Rabbit IgG (AbCam) for EpCam, Rat IgG2a (eBioscience) for CD4, and Rat IgG2b (BD Pharmingen) for CD45. Images were collected with a Zeiss Axioskop 2 FS mot confocal microscope. Images were assembled using ImageJ software.

### Gene array analysis of CD200 mRNA expression

Wt and IFNβ1^-/-^ bone marrow derived macrophages (BM-DM) were derived from C57/BL6 mice as previously described [54] and grown in 24 well plates. After 7 days of culture, BM-DM were infected with wt-MCMV, MCMV_Δ_IE3 (MOI = 1) or mock infected [55]. Cells were then harvested at 2, 4, 6, 8, 10 and 24 hours post-infection for the isolation of RNA using an RNeasy Mini kit (Qiagen, UK) according to manufacturer’s instructions. After QC using an Agilent Bioanalyzer, total RNA was labeled and hybridized to Mouse Gene 1.0ST microarrays (Affymetrix, CA, USA) according to manufacturer’s instructions using a WT Expression kit (Ambion, UK). After data capture, quality control metrics were assessed using Affymetrix Expression Console software and then all arrays were imported into Partek Genomics Suite (Partek, USA) for downstream analysis. In brief, arrays were normalized using the gcRMA algorithm [56]. After normalization, to increase confidence in the genes taken forward to statistical analysis, data was filtered to include genes with at least 1 signal value of > = 150 across the time course.

### Statistics

For viral load analysis, statistical significance was determined using the Mann-Whitney *U* test for paired groups. To analyze viral load data from bone marrow chimeras, linear regression analysis was utilized. Data were first subject to square-root transformation to introduce stability. We then fitted a linear model for covariates (donor + recipient) with and without the interaction term. Subsequent ANOVA analysis of these models showed the interaction term not to be significant (p = 0.13). However a model without any interactions is strongly significant (p = 0.0015) and was therefore used. For paired analysis of flow cytometry data, the two-tailed Student’s *t* test was utilized. For bone marrow chimeras, linear regression of non-transformed data was used. *p<0.05, **p<0.01, ***p<0.001.

### Gene accession numbers

mCD200–17470; mCD200R- 57781l; IFNγ- 15978; mIL-10–16153; mCD69–12515; mCD31/PECAM-1–18613; mIFNB1–15977; mCD80–12519

## Supporting Information

S1 Fig CD200 is expressed by endothelial cells not myoepithelial cells. (A) Isotype controls. Day 7 pi SG sections were stained with: (Left) CD200 (red) and Rat IgG2a-FITC (green), and the secondary antibodies Streptavidin 555 and Alexa Fluor 488 anti-rat IgG; (Right) CD31-FITC (green) and Rat IgG2a-Biotin (red), and the secondary antibodies Streptavidin 555 and Alexa Fluor 488 anti-rat IgG. (B) Myoepithelial cells (detected by alpha-smooth muscle actin (green)) do not express CD200 (red) in the SGs at day 14 pi. Rabbit IgG and Rat IgG2a-Biotin isotype controls were used. Magnification = 63x, white scale bars = 20μm. (C) CD200 (red) and CD31-FITC (green) co-staining of endothelial cells on a wt naïve SG section show colocalization of CD200 and endothelial cells. All sections were counterstained with TOTO-3 (blue) to detect DNA. Magnification = 63x, white scale bars = 20μm.
(TIFF)

S2 Fig Endothelial cells express CD200 during MCMV persistence and restrict SG-APC accumulation. (A) SG-APCs in wt and CD200R^-/-^ mice were enumerated 48 days post MCMV infection. (B) Wt mice were infected with MCMV and SGs harvested 14 days pi. MHC II (green) expressing cells adjacent to large CD200^+^ (red) endothelial cells are shown. Sections were counterstained with TOTO-3 (blue) to detect DNA. Magnification = 63x, white scale bars = 20μm. (C&D) Mixed wt/CD200^-/-^ bone marrow chimeras were generated and infected with MCMV. After 14 days, SG-APCs (C) and splenic DCs (D) were quantified. Individual mice + mean are shown.
(TIFF)

S3 Fig CD69^+^ CD4 T cells are enriched in CD200R^-/-^ mice during MCMV persistence. Wt and CD200R^-/-^ mice were infected with MCMV, and CD69 (A) and CD25 (B) expression by CD4 T cells from the SGs (A&B) and spleen (A) was determined 30 days pi. % expression of individual mice + mean is shown.
(TIFF)

S4 Fig CD200R does not influence MCMV replication in macrophages, or MHC II expression by macrophages. (A) Wt and CD200R^-/-^ BM-DMs were infected with MCMV (MOI: 0.5) and MCMV in supernatants were quantified by plaque assay after 6 days. Median + range is shown. (B) Representative plots from 2 experiments of F4/80 and MHC class II expression by wt (top) and CD200R^-/-^ (bottom) BM-DMs 24 hours after MCMV infection.
(TIFF)
