# Supplementary figures and images for: CD200 Receptor Restriction of Myeloid Cell Responses Antagonizes Antiviral Immunity and Facilitates Cytomegalovirus Persistence within Mucosal Tissue
Source: PLoS Pathog. 2015 Feb 5;11(2):e1004641. doi: 10.1371/journal.ppat.1004641 (PMC4412112; doi:10.1371/journal.ppat.1004641)

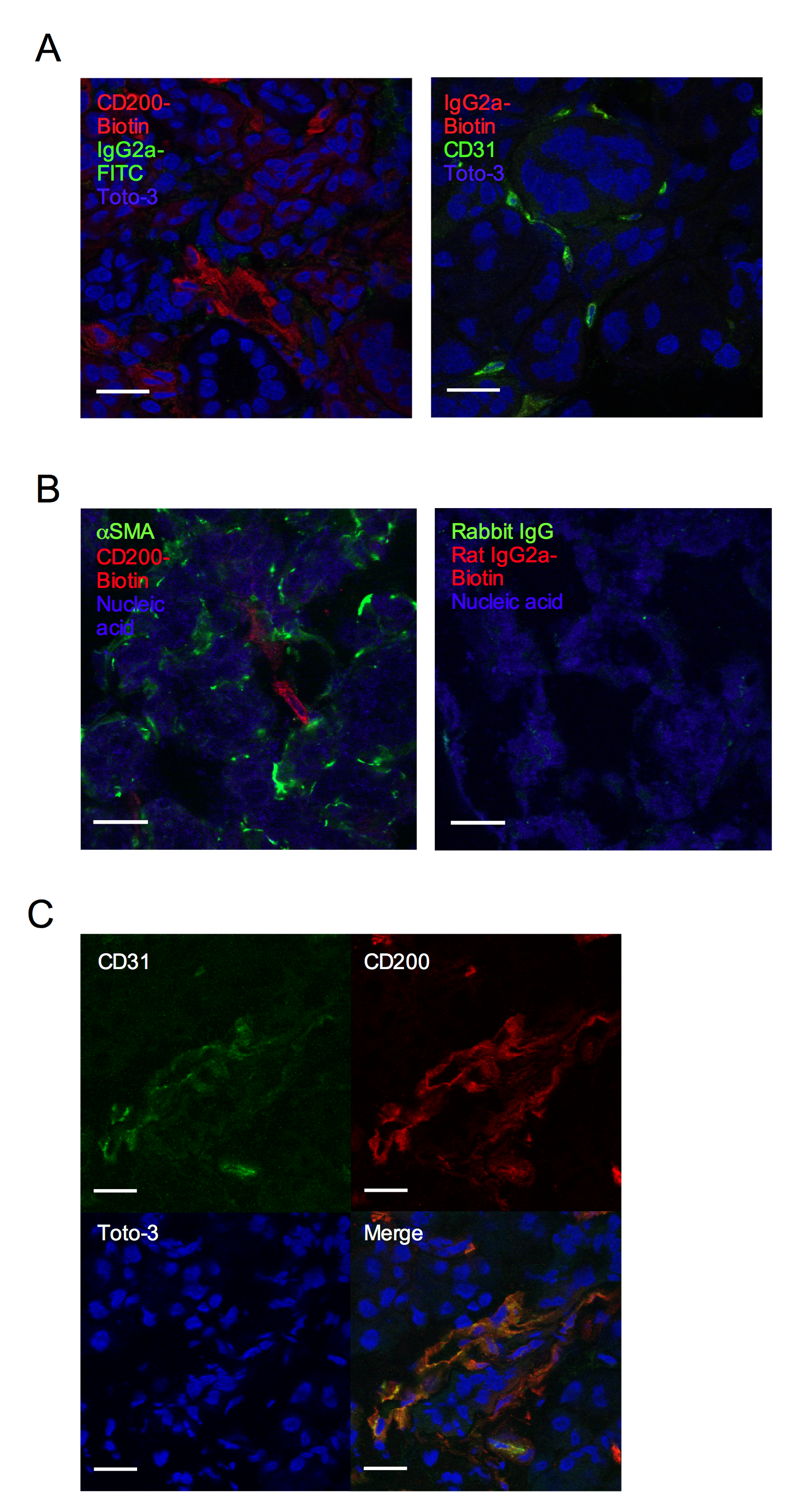

Supplement: S1 Fig — (A) Isotype controls. Day 7 pi SG sections were stained with: (Left) CD200 (red) and Rat IgG2a-FITC (green), and the secondary antibodies Streptavidin 555 and Alexa Fluor 488 anti-rat IgG; (Right) CD31-FITC (green) and Rat IgG2a-Biotin (red), and the secondary antibodies Streptavidin 555 and Alexa Fluor 488 anti-rat IgG. (B) Myoepithelial cells (detected by alpha-smooth muscle actin (green)) do not express CD200 (red) in the SGs at day 14 pi. Rabbit IgG and Rat IgG2a-Biotin isotype controls were used. Magnification = 63x, white scale bars = 20μm. (C) CD200 (red) and CD31-FITC (green) co-staining of endothelial cells on a wt naïve SG section show colocalization of CD200 and endothelial cells. All sections were counterstained with TOTO-3 (blue) to detect DNA. Magnification = 63x, white scale bars = 20μm. (TIFF) [file ppat.1004641.s001.tiff]

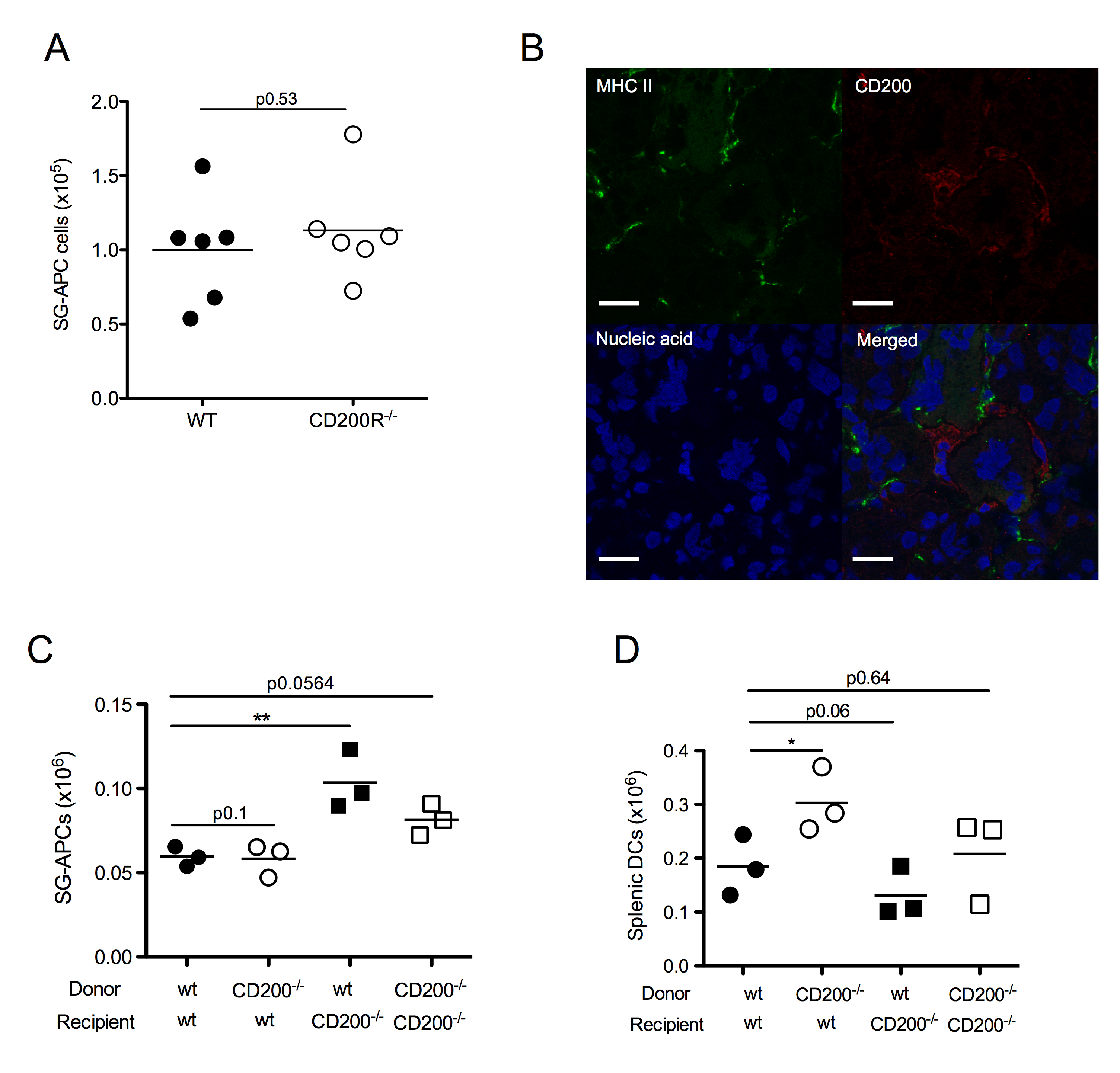

Supplement: S2 Fig — (A) SG-APCs in wt and CD200R-/- mice were enumerated 48 days post MCMV infection. (B) Wt mice were infected with MCMV and SGs harvested 14 days pi. MHC II (green) expressing cells adjacent to large CD200+ (red) endothelial cells are shown. Sections were counterstained with TOTO-3 (blue) to detect DNA. Magnification = 63x, white scale bars = 20μm. (C&D) Mixed wt/CD200-/- bone marrow chimeras were generated and infected with MCMV. After 14 days, SG-APCs (C) and splenic DCs (D) were quantified. Individual mice + mean are shown. (TIFF) [file ppat.1004641.s002.tiff]

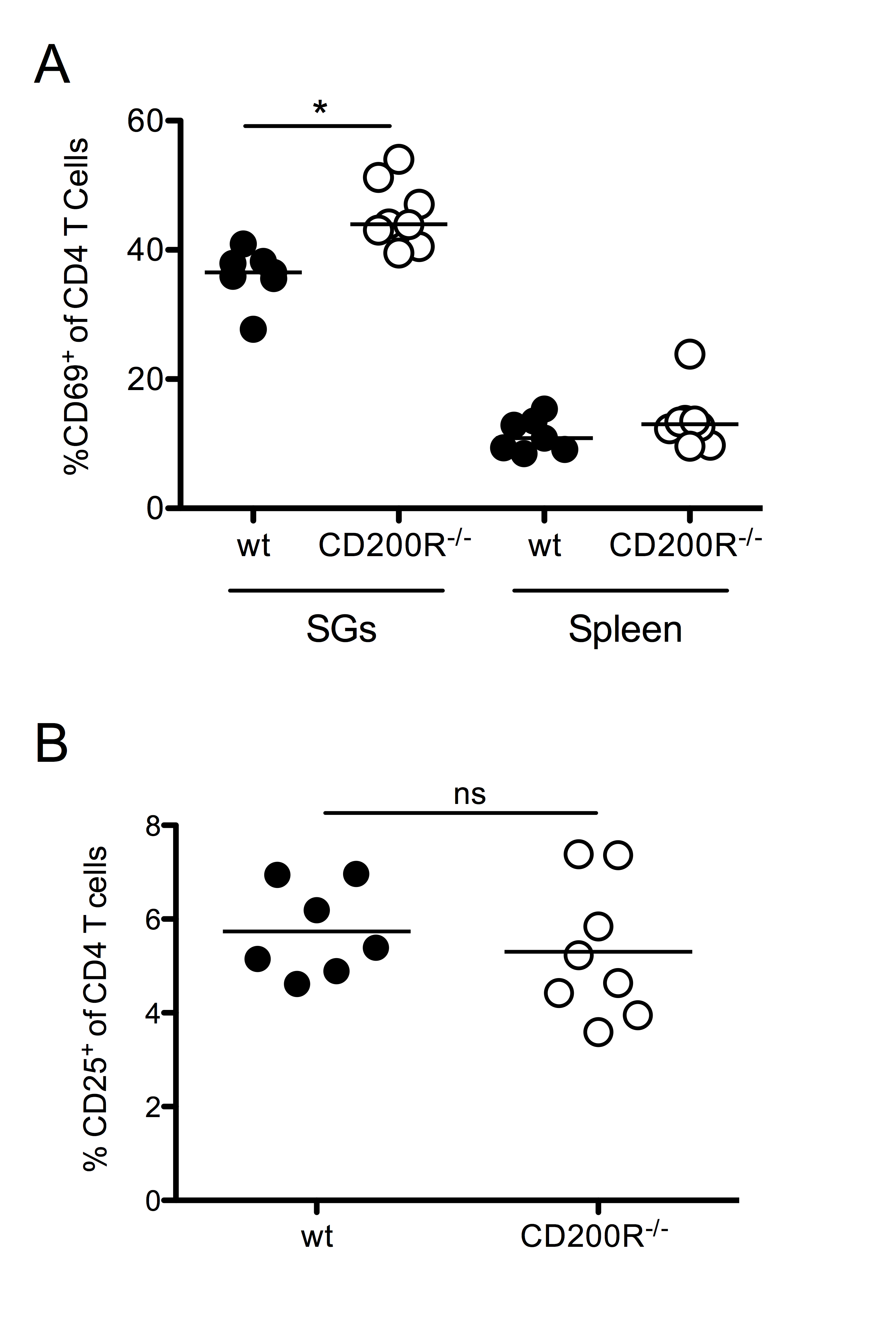

Supplement: S3 Fig — Wt and CD200R-/- mice were infected with MCMV, and CD69 (A) and CD25 (B) expression by CD4 T cells from the SGs (A&B) and spleen (A) was determined 30 days pi. % expression of individual mice + mean is shown. (TIFF) [file ppat.1004641.s003.tiff]

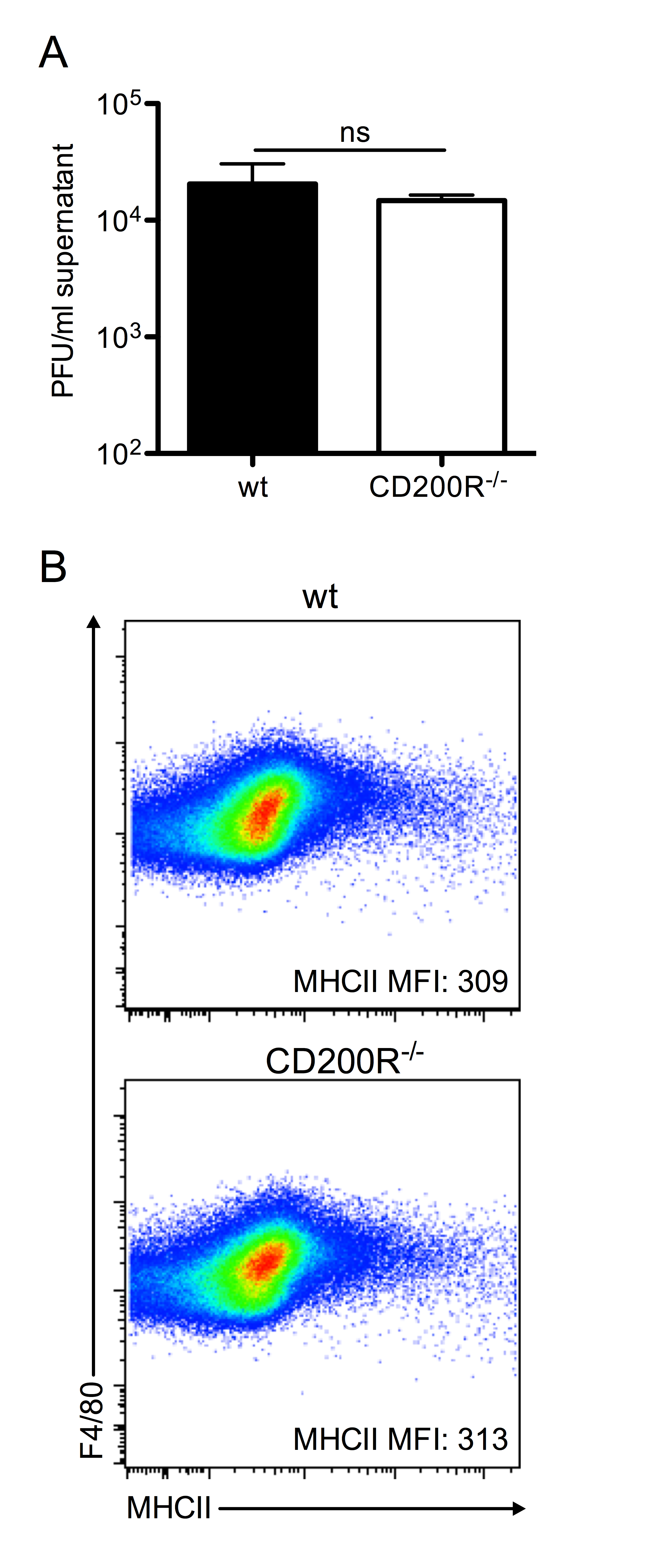

Supplement: S4 Fig — (A) Wt and CD200R-/- BM-DMs were infected with MCMV (MOI: 0.5) and MCMV in supernatants were quantified by plaque assay after 6 days. Median + range is shown. (B) Representative plots from 2 experiments of F4/80 and MHC class II expression by wt (top) and CD200R-/- (bottom) BM-DMs 24 hours after MCMV infection. (TIFF) [file ppat.1004641.s004.tiff]
